# ADHD in Prisoners

**DOI:** 10.1192/j.eurpsy.2022.73

**Published:** 2022-09-01

**Authors:** Y. Ginsberg

**Affiliations:** Karolinska Institutet, Department Of Clinical Neuroscience, Centre For Psychiatry Research, Stockholm, Sweden

**Keywords:** adhd, Prison, Treatment, Prevalence

## Abstract

The risk rate of criminality is increased in ADHD, especially in children who, in addition to ADHD, express externalizing behavior of oppositional defiant disorder (ODD), later followed by conduct disorder (CD), substance misuse and antisocial personality disorder (ASPD). Studies report ADHD to be about ten times more common in prison populations than in the general adult population. Prisoners with ADHD have compared to prisoners without ADHD, an earlier onset of offending, higher rates of coexistent psychiatric disorders, and are more often incarcerated due to violent- and drug-related offences. Within prison settings, inmates with ADHD are more often reported for intra-institutional aggression and they are often experienced as more difficult to manage and costly to rehabilitate. Further, they relapse comparably more often and faster into criminality after being conditionally released. Despite high prevalence rates of ADHD within prisons and serious consequences related to untreated ADHD, few controlled trials have evaluated methylphenidate treatment in prisoners with ADHD and coexistent disorders. Evidence and clinical experience of pharmacological and psychosocial interventions of prison populations with ADHD will be presented briefly.

**Disclosure:**

No significant relationships.

